# Clinical situations for which 3D printing is considered an appropriate representation or extension of data contained in a medical imaging examination: pediatric congenital heart disease conditions

**DOI:** 10.1186/s41205-023-00199-3

**Published:** 2024-01-29

**Authors:** Justin R. Ryan, Reena Ghosh, Greg Sturgeon, Arafat Ali, Elsa Arribas, Eric Braden, Seetharam Chadalavada, Leonid Chepelev, Summer Decker, Yu-Hui Huang, Ciprian Ionita, Joonhyuk Lee, Peter Liacouras, Jayanthi Parthasarathy, Prashanth Ravi, Michael Sandelier, Kelsey Sommer, Nicole Wake, Frank Rybicki, David Ballard

**Affiliations:** 1 Webster Foundation 3D Innovations Lab, Rady Children’s Hospital-San Diego, San Diego, CA USA; 2 Department of Neurological Surgery, UC San Diego Health, La Jolla, CA USA; 3 Department of Cardiac Surgery, Boston Children’s Hospital, Boston, MA USA; 4 Duke Children’s Pediatric & Congenital Heart Center, Durham, NC USA; 5 Department of Radiology, University of Cincinnati College of Medicine, Cincinnati, OH USA; 6 Department of Breast Imaging, The University of Texas MD Anderson Cancer Center, Houston, TX USA; 7 Arkansas Children’s Hospital, Little Rock, AR USA; 8 Joint Department of Medical Imaging, University of Toronto, Toronto, ON Canada; 9 Department of Radiology, University of South Florida Morsani College of Medicine, Tampa, USA; 10 Tampa General Hospital, Tampa, FL USA; 11 Department of Radiology, University of Minnesota, Minneapolis, MN USA; 12 Department of Biomedical Engineering, University at Buffalo, Buffalo, NY USA; 13 Department of Radiology, Walter Reed National Military Medical Center, Bethesda, MD USA; 14 Department of Radiology, Nationwide Children’s Hospital, Columbus, OH USA; 15 Department of Radiology - Advanced Reality Lab, James A. Haley VA Hospital, Tampa, FL USA; 16 Canon Medical Informatics, Minnetonka, MN USA; 17 Research and Scientific Affairs, GE HealthCare, New York, NY USA; 18 Center for Advanced Imaging Innovation and Research (CAI2R) and Bernard and Irene, Schwartz Center for Biomedical Imaging, Department of Radiology, NYU Langone Health, NYU Grossman School of Medicine, New York, NY USA; 19 Department of Radiology, University of Arizona, Phoenix, AZ USA; 20 Mallinckrodt Institute of Radiology, Washington University School of Medicine, Saint Louis, MO USA

**Keywords:** 3D printing, Appropriateness, Quality, Radiology, Additive manufacturing, Anatomic model, Cardiac, And congenital heart disease

## Abstract

**Background:**

The use of medical 3D printing (focusing on anatomical modeling) has continued to grow since the Radiological Society of North America’s (RSNA) 3D Printing Special Interest Group (3DPSIG) released its initial guideline and appropriateness rating document in 2018. The 3DPSIG formed a focused writing group to provide updated appropriateness ratings for 3D printing anatomical models across a variety of congenital heart disease. Evidence-based- (where available) and expert-consensus-driven appropriateness ratings are provided for twenty-eight congenital heart lesion categories.

**Methods:**

A structured literature search was conducted to identify all relevant articles using 3D printing technology associated with pediatric congenital heart disease indications. Each study was vetted by the authors and strength of evidence was assessed according to published appropriateness ratings.

**Results:**

Evidence-based recommendations for when 3D printing is appropriate are provided for pediatric congenital heart lesions. Recommendations are provided in accordance with strength of evidence of publications corresponding to each cardiac clinical scenario combined with expert opinion from members of the 3DPSIG.

**Conclusions:**

This consensus appropriateness ratings document, created by the members of the RSNA 3DPSIG, provides a reference for clinical standards of 3D printing for pediatric congenital heart disease clinical scenarios.

**Supplementary Information:**

The online version contains supplementary material available at 10.1186/s41205-023-00199-3.

## Introduction

Congenital heart diseases (CHD) are the most common significant birth defects [1]. 3D printing can improve patient outcomes through precise preoperative understanding of patient-specific congenital lesions. With more detailed anatomic information, clinical teams can optimize preprocedural patientcare, potentially impacting operative time, cardiopulmonary bypass time, time under anesthesia, hospital length of stay, etc. However, the added benefits of the presurgical aid may differ based on the specific clinical scenarios (i.e., surgeons may benefit from a 3D print for clinical scenario *x* over clinical scenario *y*). The Radiological Society of North America’s (RSNA) 3D Printing Special Interest Group (3DPSIG) provided an initial appropriateness rating document in 2018 which encapsulated congenital heart disease along with other non-cardiac clinical scenarios [2]. The purpose of this document is to update the clinical indications for 3D printing in congenital heart disease, and then vet, vote, and publish recommendations on their appropriateness.

There are numerous methods of classifying CHD lesions (e.g., by STAT category, by intended surgical treatment, by fetal development, etc.). The recommendations in this document largely conform to the CHD nomenclature defined by the European Association for Cardio-Thoracic Surgery / Society of Thoracic Surgery (EACTS-STS) version of the International Pediatric and Congenital Cardiac Code.

## Methods

The 3DPSIG developed a Congenital Heart Disease Conditions Group to review the clinical scenarios for which 3D Printing is considered appropriate. This document follows the American College of Radiology (ACR) Appropriateness Criteria® construct regarding the scoring of publications as evidence-based medicine [3]. When insufficient evidence is obtained (largely through structured PubMed searches), expert consensus from the voluntary 3D Printing Congenital Heart Disease Conditions Voting Group is utilized to determine appropriate use.

The 3DPSIG’s Appropriateness Ratings Chairpersons defined the ratings of appropriate use for clinical scenarios to follow the following 1–9 format (with 9 being the most appropriate):

- 1–3, rarely appropriate (red). There is a lack of a clear benefit or experience that shows an advantage over usual practice.
- 4–6, maybe appropriate (yellow). There may be times when there is an advantage, but the data is lacking, or the benefits have not been fully defined.
- 7–9, usually appropriate (green). Data and experience show an advantage to 3D printing as a method to represent and/or extend the value of data contained in the medical imaging examination.

The search results were reviewed by experts and some references were removed and some were relocated to different categories. As noted above, references outside of the structured searches were added but noted and approved by the writing group. As a general rule, the benefits of 3D printing to define and rehearse an intervention increase with the overall degree of disease complexity.

An exhaustive US National Library of Medicine (PubMed) literature search was performed (publications dated July 22, 2022 or earlier were included) which enabled the querying and retrieval of appropriate clinical documents supporting the appropriateness of 3D printing-enabled technologies for each specific diagnosis. For each clinical scenario, the PubMed query results were reviewed for relevancy to 3D printing and CHD. Publication exclusion criteria included articles with complete subject mismatch (e.g., results for atrial septum defects were often conflated with results for autism spectrum disorder due to the shared “ASD” acronym), non-English language publications (to limit errors-in-translations from affecting impact of appropriateness ratings), and publications out of the technological and/or patient-care scope (e.g., publications focusing on extended reality, education, bioprinting, anthropomorphic models, phantoms, etc.). The remaining publications [4–58] were reviewed by senior authors following the ACR Appropriateness Criteria® construct for reviewing evidence-based medicine.

All clinical scenarios were vetted and approved by the 3D Printing Congenital Heart Disease Conditions Voting Group virtually at the July 22, 2022 and September 21, 2022 3DPSIG teleconference calls. Members largely composed of physicians (primarily radiologists), scientists, technologists, biomedical engineers, and other 3D printing subject matter experts. All votes and ratings were unanimous unless otherwise noted. The ratings were posted in the 3DPSIG’s member only site for further comments and discussion. The paper represents the findings and conclusions of the 3DPSIG and does not represent an endorsement by the Radiological Society of North America (RSNA).

## Results

Table 1 provides a summary of evidence-based and expert consensus to define and support the use of 3D printing for patients with CHD. The citations included in forming the appropriateness recommendations and the strength of evidence assessment are presented in Appendices 1 and 2 respectively. Studies from the structured literature search that had no direct patient care impact and were excluded from being used in the appropriateness ratings are presented in Appendix 3.

**Table 1:**
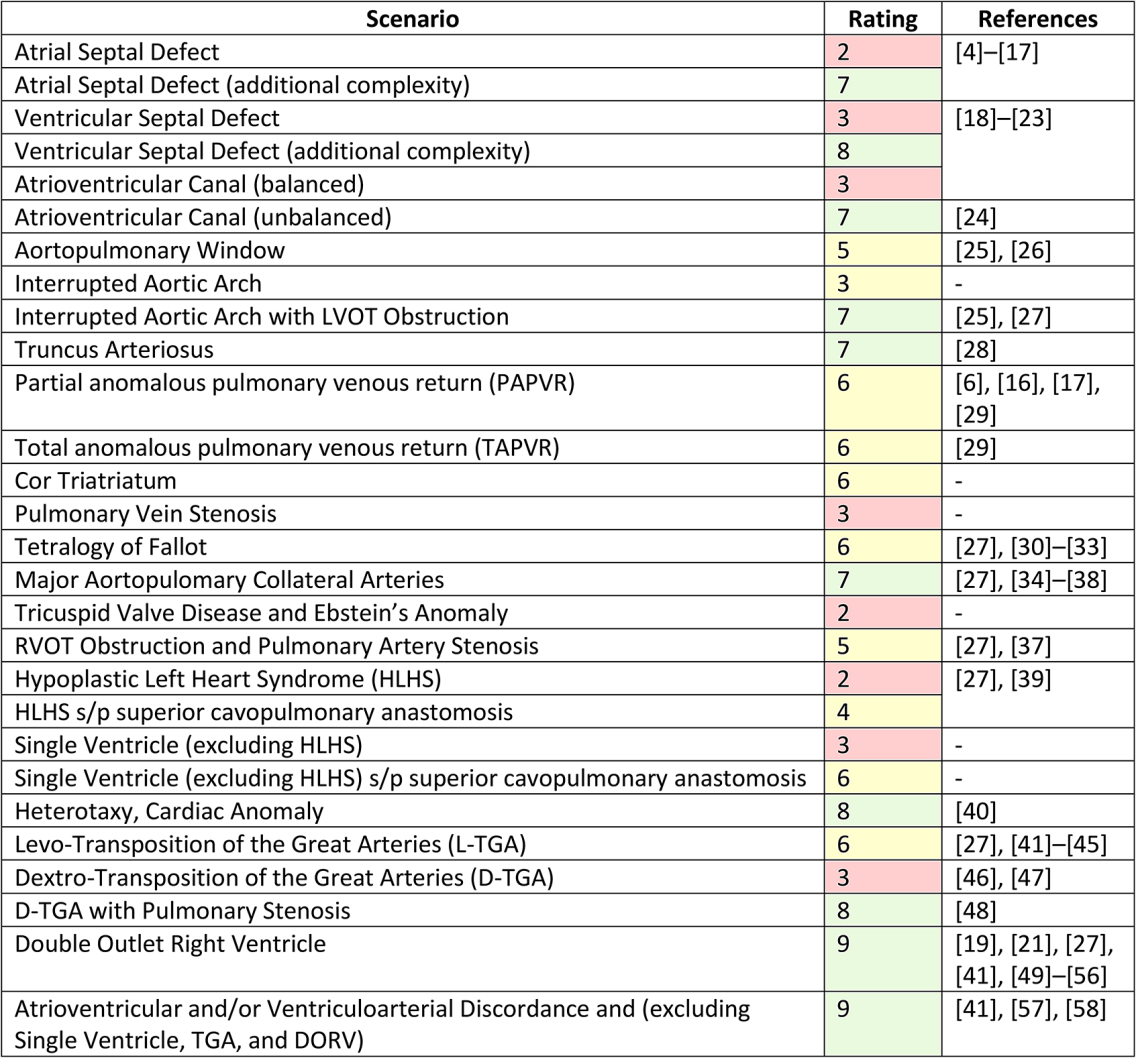
Appropriateness ratings for patients with congenital heart disease conditions

## Discussion

CHD lesions have a wide range of presentations as well as surgical/interventional palliations. There was an attempt to capture some major variations in lesions that would follow different surgical pathways as a separate clinical scenario (e.g., D-TGA and D-TGA with pulmonary stenosis). There was also an effort to group lesions with similar surgical pathways under a common broader category (e.g., Single Ventricle (excluding HLHS) to include double inlet left ventricle and tricuspid atresia). Encompassing the entire spectrum of possible lesions and operations and determining 3D printing appropriateness definitively is not feasible.

### Atrial septal defect (ASD)

An atrial septal defect is a hemodynamic lesion resulting in pathologic communication between the right and left atrium. Without further modifiers, this term refers to primum or secundum septal defects. There is no literature to support 3D printing for primum or secundum ASDs without additional complexity.

Compared to the prior 2018 rating [2], ASD was changed from size-based classification to categorization by complexity. ASD rating remained red (Table 1).

### Atrial septal defect (additional complexity)

The “additional complexity” modifier refers to atrial septal defects excluding typical presentations of primum or secundum presentations. Typically, this category refers to a defect in the sinus venosus component of the atrial septum, that results in a pathologic communication between the right and left atrium in conjunction with anomalous systemic or pulmonary venous drainage. Less commonly, this can also describe a coronary sinus septal defect (unroofed coronary sinus) as well as device evaluation in secundum ASDs with deficient rims [4, 7, 15] or in the presence of multiple ASDs [14]. There is substantial qualitative support [4–17] for 3D printing for ASDs with additional complexity especially for interventional planning.

“ASD, Large” in the prior document had a red rating now amended to green for ASD (additional complexity) (Table 1). There was consensus in the rating; however, a minority vote (2/20 GS, NW) recommended separating ASD types by anatomic location.

### Ventricular septal defect (VSD)

A ventricular septal defect is an absence of tissue in one or more of the four components of the ventricular septum (membranous, inlet, muscular/trabecular, infundibulum). VSDs (without additional complexity) are not perceived by expert consensus to benefit from a 3D print.

### Ventricular septal defect (additional complexity)

The “additional complexity” modifier refers to defects which are large and extend into more than one component of the ventricular septum, defects with associated AV valve tissue, or the presence of multiple VSDs. Examples include multiple muscular VSDs (including what has been colloquially referred to as “swiss-cheese septum”) or an inlet VSD with straddling tricuspid valve. Literature [19, 21–23] and expert consensus support 3D printing especially for interventional planning or when complex patches are needed [18].

Compared to the prior 2018 rating, VSD was changed from size-based classification to categorization by complexity in order to provide guidance across a wide range of pathology. VSD (additional complexity) had prior yellow rating amended to green (Table 1). The recommendation passed by a majority vote (18/20 votes); the two voting members that dissented (GS, NW) advocated for classifying VSD types by anatomical location.

### Atrioventricular canal (balanced)

An atrioventricular canal (balanced) is an endocardial cushion defect resulting in a single large atrioventricular valve that provides equal inflow into both ventricles, in the setting of a primum ASD and inlet VSD. Variants exist such as a transitional AV canal (primum ASD with a restrictive inlet VSD), incomplete AV canal (large inlet VSD with no primum ASD component) or partial AV canal (primum ASD with no inlet VSD and a cleft left AV valve). There is insufficient literature or expert consensus to support 3D printing for balanced AV Canals.

### Atrioventricular canal (unbalanced)

An unbalanced AV canal is an endocardial cushion defect resulting in a single large atrioventricular valve that provides inflow primarily to one ventricle and not the other. This can result in hypoplasia of the ventricle that receives less blood flow. While there are limited publications supporting 3D printing for unbalanced AV Canals [24], expert consensus strongly supported 3D printing to assist with complex surgical patch planning.

### Aortopulmonary window

An aortopulmonary (AP) window is an incomplete development of the conotruncal septum resulting in a pathologic connection between the ascending aorta and either the main or right pulmonary artery. Limited publications [25, 26] support 3D printing with the later publication discussing the use of 3D printing to decide between intervention and surgery. Expert consensus agrees that 3D printing is sometimes appropriate for AP Windows.

### Interrupted aortic arch

An interrupted aortic arch (IAA) includes atresia of any component of the aortic arch, which results in ductal-dependent systemic blood flow. This initial term does not include (IAA) defects presenting with left ventricular outflow (LVOT) obstruction. Expert consensus in the paucity of literature agrees that 3D printing is rarely appropriate without confounding factors.

### Interrupted aortic arch with LVOT obstruction

Atresia as listed above, with additional narrowing of the left ventricular outflow tract. The narrowing is often caused by posterior malalignment of the conal septum (with or without aortic valve hypoplasia) with an accompanying VSD. A single case in a case series [27] and expert consensus concur that 3D printing for this lesion can be beneficial in surgical planning for intracardiac pathways.

Compared to the prior 2018 rating, IAA characterization was refined by separating the IAA lesion into two clinical scenarios. IAA with LVOT obstruction was given a green rating.

### Truncus arteriosus

Truncus arteriosus is a conotruncal anomaly resulting in a common outflow trunk giving rise to both the aortic arch and pulmonary arteries. The variations of truncus arteriosus are related to the anatomic configuration of the aortic arch, head and neck vessels and origin of the right and left pulmonary arteries. A case study [28] and expert consensus concur that 3D printing for this lesion can be beneficial in surgical planning for intracardiac pathways.

### Total anomalous pulmonary venous return (TAPVR)

Total anomalous pulmonary venous return/connection (TAPVR or TAPVC) is a failure of the posterior left atrium to fuse with the pulmonary venous plexus, resulting in a lack of connection between all of the pulmonary veins and the left atrium. Instead, the pulmonary veins drain into a systemic venous structure. This defect can be characterized as supracardiac (i.e., pulmonary venous return to a vertical vein), cardiac (i.e., to the right atrium), infracardiac (i.e., to the IVC as in Scimitar syndrome) or mixed (pulmonary venous connections to more than one systemic venous structure). A single case series [29] in which 14 out of 17 patients had TAPVR described surgical planning with 3D prints and discussed a particular strength in using the anatomic model for follow-up care; however, experts agreed that 3D printing for TAVPR cases is only sometimes appropriate given other imaging modalities.

The rating was amended from green to yellow in the most recent voting session. The voting group approved the rating with a majority vote (19/20 votes); the one voting member that dissented from the rating (GS) wanted a lower rating. The member’s perspective is that TAPVR lesions are generally well characterized by other modalities.

### Partial anomalous pulmonary venous return (PAPVR)

Partial anomalous pulmonary venous return/connection is an aberrant drainage of a portion of the pulmonary venous return to a systemic venous structure. This is often associated with a sinus venosus ASD. Literature and expert consensus for PAPVR lesions was similar to that of TAPVR lesions regarding efficacy of 3D printing.

The rating was amended from green to yellow in the most recent voting session.

### Cor triatriatum

Cor Triatriatum is the presence of an additional membrane in either the right or left atrium. Most often seen in the left atrium, the membrane separates the proximal (pulmonary venous) chamber from the distal left atrium (including the left atrial appendage), which communicates with the mitral valve. Severity of symptoms depend on the presence or absence of egress between the proximal and distal chambers and/or other shunting lesions. While no literature was obtained through the structured PubMed search, expert consensus agreed that 3D printing was sometimes appropriate – especially for planning complex interventional procedures.

### Pulmonary vein stenosis

Pulmonary vein stenosis is defined by intimal hyperplasia of the pulmonary veins leading to varying degrees of luminal narrowing. Acquired or secondary pulmonary vein stenosis can occur after surgical or transcatheter intervention on the pulmonary veins; however, acquired disease is out of the scope of this document. At this time, no studies have been performed suggesting the utility of 3D printing in such cases.

### Tetralogy of fallot

Tetralogy of Fallot (ToF) is a constellation of intracardiac lesions resulting from anterior deviation of the conal septum. These lesions are (1) VSD, (2) pulmonary stenosis, (3) right ventricular hypertrophy, and (4) overriding aorta. There is a wide range of symptomatology based on the degree of pulmonary stenosis, which can range from minimal to pulmonary atresia, with neonatal ductal dependent pulmonary blood flow. 3D printing may have limited support for spatial relationship between the ventricular septal defect, tricuspid valve, and the RVOT [31–33]. 3D printing has been utilized on planning of percutaneous pulmonary valve replacement in repaired Tetralogy of Fallot patients or ductal stenting in neonates with ductal dependent pulmonary blood flow [59]. The rating remained yellow by a majority vote (19/20 votes); the one voting member (GS) advocated for a lower rating.

### Major aortopulmonary collateral arteries

Major aortopulmonary collateral arteries are vessels that arise from the aorta and supply blood flow to the pulmonary arteries and/or directly supply segments of the lung. They are typically associated with lesions such as Tetralogy of Fallot/Pulmonary Atresia, but can be found in any pathology resulting in a lack of pulmonary blood flow.

Beyond the benefits described above, 3D printing is beneficial in appreciating the highly differential morphology as well as in planning of unifocalization according to case studies and series [27, 34–38].

### Tricuspid valve disease and ebstein’s anomaly

Tricuspid valve disease and Ebstein’s anomaly encompass a wide range of tricuspid valve pathology resulting in either stenosis, regurgitation, or mixed valve disease. Ebstein’s anomaly is a specific lesion characterized by apical displacement of the septal and posterior leaflets and a flail anterior leaflet resulting in varying degrees of tricuspid regurgitation. There is often accompanying atrialization of the basal component of the right ventricle. The associated rating was derived solely from expert consensus.

### RVOT obstruction and pulmonary artery stenosis

The combined clinical scenario includes right-sided lesions that can impede blood flow to the lungs. Right ventricular outflow tract (RVOT) obstruction is typically due to hypertrophied muscle bundles in the RV infundibulum or anterior deviation of the conal septum. Pulmonary artery stenosis encompasses a wide range of pathology including supravalvar pulmonary stenosis, pulmonary artery hypoplasia, or focal stenosis of the right or left pulmonary arteries or their branches. A limited cohort of cases from case series along with expert consensus support 3D printing for surgical and interventional planning and management [27, 37].

### Hypoplastic left heart syndrome (HLHS)

Hypoplastic left heart syndrome (HLHS) is defined by an underdevelopment of left heart structures that cannot support systemic circulation. This is characterized by mitral and aortic valve stenosis or atresia, in the setting of an intact ventricular septum. These patients require a procedure in the neonatal period to either maintain ductal flow (PDA stent) or create a neo-aortic valve to provide systemic output along with placement of a systemic to pulmonary shunt (commonly known as a “Stage 1 palliation”). 3D printing is purported to support morphologic understanding of this disease process, especially for complex, patient-specific surgeries [27, 39].

Compared to the prior 2018 rating, HLHS was recategorized to reflect different clinical scenarios: “HLHS (covering planning of initial palliation and stage 2 palliation)” and “HLHS s/p superior cavopulmonary anastomosis (stage 2 palliation).”

There was consensus on updating the rating of the new HLHS category from yellow to red in the most recent voting session. However, a minority (1/20 votes) advocated for each stage of palliation to be considered as a separate clinical scenario.

### HLHS s/p superior cavopulmonary anastomosis

HLHS s/p superior cavopulmonary anastomosis refers to patients with HLHS after a surgical connection is created from the superior vena cava (SVC) to the right pulmonary artery (RPA). This provides passive venous return from the upper body to the pulmonary vascular bed. In conjunction with the removal of the systemic to pulmonary shunt placed in the neonatal period, this surgical repair is commonly known as a “Stage 2 palliation”. Expert consensus elevated the rating for the subset of HLHS given ability of 3D printing to illustrate the complex anatomy created by the superior cavopulmonary anastomosis.

### Mitral atresia

Mitral atresia was removed as a separate clinical scenario and is now reflected under HLHS.

### Tricuspid atresia

Tricuspid atresia was removed as a separate clinical scenario and is now reflected under Single Ventricle (excluding HLHS).

### Shone’s syndrome

Shone’s Syndrome was removed as a separate clinical scenario in the most recent rating.

### Single ventricle (excluding HLHS)

Single ventricle (excluding HLHS) refers to a variety of pathologies that result in only one functional ventricle. Regardless of the native anatomy, patients with these lesions cannot support pulmonary and systemic circulations in series. Examples of pathologies that result in single ventricle physiology are tricuspid atresia, unbalanced atrioventricular canal, double inlet left ventricle, etc. The rating for single ventricle (excluding HLHS) was supported by expert consensus given the lack of literature discovered in the structured PubMed query.

Compared to the prior 2018 rating, “Single Ventricle (general)” was recategorized to reflect different clinical scenarios: “Single Ventricle (excluding HLHS)” and “Single Ventricle (excluding HLHS) s/p superior cavopulmonary anastomosis” The voting group concurred with a majority vote (19/20 votes); the one voting member that dissented from the categories (GS) advocated for each stage of palliation to be considered as a separate clinical scenario.

### Single ventricle (excluding HLHS) s/p superior cavopulmonary anastomosis

Single ventricle (excluding HLHS) s/p superior cavopulmonary anastomosis refers to patients with native anatomy as described above, who undergo surgical creation of a connection between the superior vena cava and right pulmonary artery to facilitate passive pulmonary blood flow.

### Heterotaxy, cardiac anomaly

Heterotaxy syndrome is characterized by abnormal orientation of the thoracoabdominal viscera along the right-left axis. Commonly, the constellation of cardiac lesions seen in heterotaxy are categorized based on the appearance of the spleen. In heterotaxy, asplenia-type, patients frequently have a complete AV canal, TAPVR, bilateral SVCs and ventriculoarterial anomalies. In heterotaxy, polysplenia-type, patients frequently have bilateral SVCs, interruption of the IVC and PAPVR. Due to the highly variable cardiac and surrounding anatomy, expert consensus largely drove the associated rating; a single case study supported the rating [40].

### Levo-transposition of the great arteries (L-TGA)

Levo-Transposition of the Great Arteries is an anomaly characterized by ventriculoarterial discordance in patients with L-looped ventricles. Often referred to as congenitally corrected transposition of the great arteries, patients with this anatomy have normal cardiovascular physiology. There is a variant of L-TGA in which the morphologic right ventricle is hypoplastic, resulting in single ventricle physiology. According to a series of case studies and case series [27, 41, 42, 44, 45] as well as expert consensus, 3D printing supports morphologic understanding and facilities complex baffle planning (two publications were captured in the DTGA search query, but case descriptions confirm LTGA anatomy [27, 45]). One case study even supported using 3D printing along with complementary modalities, and the associated morphologic understanding, to schedule (defer) surgery [43].

Compared to the 2018 rating, L-TGA is now used for the nomenclature instead of Congenitally Corrected-TGA. The rating was amended from green to yellow.

### Dextro-transposition of the great arteries (D-TGA)

Dextro-Transposition of the Great Arteries is an anomaly characterized by ventriculoarterial discordance in patients with D-looped ventricles. Patients with this pathology are born with the systemic and pulmonary circulation in parallel, rather than in series. The most commonly performed surgical repair of this lesion is the arterial switch operation. Expert consensus and minimal literature [46, 47] support the associated red rating due to perceived lack of benefit for this patient population. Both case studies had elements of additional complexity (post-surgery) that would further complicate the intended repair.

Compared to the prior 2018 rating, “Dextro-Transposition of the Great Arteries (D-TGA)” was recategorized to reflect different clinical scenarios: “Dextro-Transposition of the Great Arteries (D-TGA)” and “D-TGA with pulmonary stenosis” D-TGA was amended from green to red.

### D-TGA with pulmonary stenosis

D-TGA with Pulmonary Stenosis commonly involves posterior deviation of the conal septum. Based on the degree of pulmonary valve hypoplasia and/or left ventricular outflow tract obstruction, these patients may not be candidates for an arterial switch operation and require surgical procedures that involve relief of the LVOTO and/or placement of an RV to PA conduit. Expert consensus strongly supported 3D printing for this patient population, a single case study also supported the use of 3D printing (for interventional planning) [48].

### Double outlet right ventricle

A conotruncal anomaly that results in both great vessels arising primarily from the right ventricle, with an accompanying VSD. This term encompasses a range of pathology, from “Tet-like” DORV where the aorta is closest to the VSD and left ventricle, as well as DORV with mitral atresia. The ability to conduct a biventricular repair is largely dependent on the relationship between the VSD and the great vessels, with particular attention paid to any intervening structures (i.e. tricuspid chordal attachments). Double outlet right ventricle pathology had the greatest literature-base support for 3D printing [19, 21, 27, 41, 49–56] with most discussions adding to the unique benefit for surgeons to plan complex intracardiac pathways.

### Double inlet left ventricle (DILV)

DILV is now reflected under “Single Ventricle (excluding HLHS).”

### Double inlet right ventricle (DIRV)

DIRV is now reflected under “Single Ventricle (excluding HLHS).”

### Atrioventricular and/or ventriculoarterial discordance (excluding single ventricle, TGA and DORV)

These terms refer to pathologies in which the atria do not primarily connect to their “corresponding” ventricle, or if the ventricles do not connect to their “corresponding” great vessel. These categories include lesions such as superior-inferior ventricles or criss-cross ventricles. Surgical repair often requires detailed understanding of the three-dimensional spatial relationships between the intracardiac structures. Limited case studies [41, 57, 58] and expert consensus support the use of 3D printing due to the highly variable presentation of cardiac structures in this patient population.

## Conclusion

This document provides updated appropriateness ratings for 3D printing in congenital heart disease. Dissemination of clinical standards regarding appropriate use, information and material management, and quality control are needed to ensure the greatest possible clinical benefit from 3D printing as well as consistency across institutions. Increased volume of anatomical modeling events, this consensus appropriateness ratings, and growing evidence for 3D printing in pediatric congenital heart disease will provide a framework for clinical standards of 3D printing in medicine. This consensus appropriateness ratings will be periodically reviewed and refreshed with the latest publications reflecting growth and changes in medical literature.

### Electronic supplementary material

Below is the link to the electronic supplementary material.

Supplementary Material 1: Structured PubMed search terms for each clinical scenario in the appropriateness document

Supplementary Material 2: Level of evidence for publications in the appropriateness document

Supplementary Material 3: Publications of note related to 3D technologies and congenital heart disease, but not meeting eligibility criteria for inclusion in the appropriateness rating

Supplementary Material 4: Change log detailing additions and amendments from the 2018 appropriateness ratings and this revised manuscript

## Data Availability

No datasets were generated or analysed during the current study.

## Data Availability

The datasets used and/or analyzed during the current study are available from the corresponding author on reasonable request.
