# Supplementary material for: Clinical situations for which 3D printing is considered an appropriate representation or extension of data contained in a medical imaging examination: pediatric congenital heart disease conditions
Source: 3D Print Med. 2024 Jan 29;10:3. doi: 10.1186/s41205-023-00199-3 (PMC10823658; doi:10.1186/s41205-023-00199-3)
Supplement: Supplementary file 1 — Supplementary Material 1: Structured PubMed search terms for each clinical scenario in the appropriateness document [file 41205_2023_199_MOESM1_ESM.docx]

# Appendix I: Search Terms

## Background

Structured PubMed search terms for each clinical scenario in the appropriateness document.

## Atrial Septal Defect (ASD)

**PubMed Search:** ((“printing, three-dimensional”[MeSH Terms] OR (“printing”[All Fields] AND “three-dimensional”[All Fields]) OR “three-dimensional printing”[All Fields] OR (“3d”[All Fields] AND “printing”[All Fields]) OR “3d printing”[All Fields]) OR ("rapid"[All Fields] AND "prototyping"[All Fields]) OR ("additive"[All Fields] AND "manufacturing"[All Fields])) AND ((“Heart Septal Defects, Atrial”[MeSH Terms] OR (“atrial”[All Fields] AND “septal”[All Fields] and "defect"[All Fields]) OR “atrial septal defect”[All Fields] OR “unroofed coronary sinus”[All Fields]))

* “Additional complexity” is intended to include superior sinus venous defect, Inferior sinus venous defect, and other anatomical variations that would complicate surgery and/or intervention.

**Search Results:** 37 initial results, 15 after exclusions

## Ventricular Septal Defect (VSD)

**PubMed Search:** ((“printing, three-dimensional”[MeSH Terms] OR (“printing”[All Fields] AND “three-dimensional”[All Fields]) OR “three-dimensional printing”[All Fields] OR (“3d”[All Fields] AND “printing”[All Fields]) OR “3d printing”[All Fields]) OR ("rapid"[All Fields] AND "prototyping"[All Fields]) OR ("additive"[All Fields] AND "manufacturing"[All Fields])) AND (“Heart Septal Defects, Ventricular”[MeSH Terms] OR (“ventricular”[All Fields] AND “septal”[All Fields] and "defect"[All Fields]) OR “ventricular septal defect”[All Fields] OR “VSD”[All Fields])

** “Additional complexity” is intended to include mid muscular and apical forms of VSDs and other anatomical variations that would complicate surgery and/or intervention.

**Search Results:** 64 initial results, 6 after exclusions

## Atrioventricular (AV) Canal

**PubMed Search:** ((“printing, three-dimensional”[MeSH Terms] OR (“printing”[All Fields] AND “three-dimensional”[All Fields]) OR “three-dimensional printing”[All Fields] OR (“3d”[All Fields] AND “printing”[All Fields]) OR “3d printing”[All Fields]) OR ("rapid"[All Fields] AND "prototyping"[All Fields]) OR ("additive"[All Fields] AND "manufacturing"[All Fields])) AND ((“Atrioventricular Septal Defect”[MeSH Terms] OR (“Atrioventricular”[All Fields] AND “septal”[All Fields] and "defect"[All Fields]) OR “atrioventricular canal”[All Fields] OR “av canal”[All Fields]))

**Search Results:** 7 initial results, 1 after exclusions

## Aortopulmonary (AP) Window

**PubMed Search:** ((“printing, three-dimensional”[MeSH Terms] OR (“printing”[All Fields] AND “three-dimensional”[All Fields]) OR “three-dimensional printing”[All Fields] OR (“3d”[All Fields] AND “printing”[All Fields]) OR “3d printing”[All Fields]) OR ("rapid"[All Fields] AND "prototyping"[All Fields]) OR ("additive"[All Fields] AND "manufacturing"[All Fields])) AND (“Aortopulmonary Septal Defect”[MeSH Terms] OR (“Aortopulmonary”[All Fields] AND “septal”[All Fields] and "defect"[All Fields]) OR “AP Window”[All Fields])

**Search Results:** 9 initial results, 2 after exclusions

## Interrupted Aortic Arch

**PubMed Search:** (("printing, three-dimensional"[MeSH Terms] OR ("printing"[All Fields] AND "three-dimensional"[All Fields]) OR "three-dimensional printing"[All Fields] OR ("3d"[All Fields] AND "printing"[All Fields]) OR "3d printing"[All Fields]) OR ("rapid"[All Fields] AND "prototyping[All Fields]) OR ("additive"[All Fields] AND "manufacturing"[All Fields])) AND ("Interrupted Aortic"[All Fields] OR "Interrupted Aorta"[All Fields] OR "IAA”[All Fields])

**Search Results:** 2 initial results, 1 after exclusions

## Truncus Arteriosus

**PubMed Search:** ((“printing, three-dimensional”[MeSH Terms] OR (“printing”[All Fields] AND “three-dimensional”[All Fields]) OR “three-dimensional printing”[All Fields] OR (“3d”[All Fields] AND “printing”[All Fields]) OR “3d printing”[All Fields]) OR ("rapid"[All Fields] AND "prototyping"[All Fields]) OR ("additive"[All Fields] AND "manufacturing"[All Fields])) AND ("Truncus Arteriosus"[MeSH Terms] OR "Truncus Arteriosus, Persistent"[MeSH Terms] OR (“truncus”[All Fields] AND “arteriosus”[All Fields]) OR “truncus arteriosus”[All Fields] OR “truncus”[All Fields])

**Search Results:** 1 initial results, 1 after exclusions

## Partial Anomalous Pulmonary Venous Return (PAPVR)

**PubMed Search:** ((“printing, three-dimensional”[MeSH Terms] OR (“printing”[All Fields] AND “three-dimensional”[All Fields]) OR “three-dimensional printing”[All Fields] OR (“3d”[All Fields] AND “printing”[All Fields]) OR “3d printing”[All Fields]) OR ("rapid"[All Fields] AND "prototyping"[All Fields]) OR ("additive"[All Fields] AND "manufacturing"[All Fields])) AND ("Scimitar Syndrome"[MeSH Terms] OR “partial anomalous pulmonary venous connection”[All Fields] OR “partial anomalous pulmonary venous return”[All Fields] OR “partial anomalous pulmonary venous”[All Fields] OR “anomalous pulmonary venous”[All Fields] OR “PAPVR”[All Fields] OR “PAPVC”[All Fields])

**Search Results:** 4 initial results, 4 after exclusions

## Total Anomalous Pulmonary Venous Return (TAPVR)

**PubMed Search:** ((“printing, three-dimensional”[MeSH Terms] OR (“printing”[All Fields] AND “three-dimensional”[All Fields]) OR “three-dimensional printing”[All Fields] OR (“3d”[All Fields] AND “printing”[All Fields]) OR “3d printing”[All Fields]) OR ("rapid"[All Fields] AND "prototyping"[All Fields]) OR ("additive"[All Fields] AND "manufacturing"[All Fields])) AND (“total anomalous pulmonary venous connection”[All Fields] OR “total anomalous pulmonary venous return”[All Fields] OR “total anomalous pulmonary venous”[All Fields] OR “anomalous pulmonary venous”[All Fields] OR “TAPVR”[All Fields] OR “TAPVC”[All Fields])

**Search Results:** 4 initial results, 1 after exclusions

## Cor Triatriatum

**PubMed Search:** ((“printing, three-dimensional”[MeSH Terms] OR (“printing”[All Fields] AND “three-dimensional”[All Fields]) OR “three-dimensional printing”[All Fields] OR (“3d”[All Fields] AND “printing”[All Fields]) OR “3d printing”[All Fields]) OR ("rapid"[All Fields] AND "prototyping"[All Fields]) OR ("additive"[All Fields] AND "manufacturing"[All Fields])) AND (“Cor Triatriatum”[MeSH Terms] OR (“Cor Triatriatum”[All Fields]))

**Search Results:** 0 results

## Pulmonary Vein Stenosis

**PubMed Search:** ((“printing, three-dimensional”[MeSH Terms] OR (“printing”[All Fields] AND “three-dimensional”[All Fields]) OR “three-dimensional printing”[All Fields] OR (“3d”[All Fields] AND “printing”[All Fields]) OR “3d printing”[All Fields]) OR ("rapid"[All Fields] AND "prototyping"[All Fields]) OR ("additive"[All Fields] AND "manufacturing"[All Fields])) AND (“Stenosis, Pulmonary Vein”[MESH Terms] OR “Pulmonary Vein Stenosis”[All Fields] OR “Pulmonary Vein Stenoses”[All Fields])

**Search Results:** 0 results

## Tetralogy of Fallot

**PubMed Search:** ((“printing, three-dimensional”[MeSH Terms] OR (“printing”[All Fields] AND “three-dimensional”[All Fields]) OR “three-dimensional printing”[All Fields] OR (“3d”[All Fields] AND “printing”[All Fields]) OR “3d printing”[All Fields]) OR ("rapid"[All Fields] AND "prototyping"[All Fields]) OR ("additive"[All Fields] AND "manufacturing"[All Fields])) AND (“tetralogy of fallot”[MESH Terms] OR “tetralogy of fallot”[All Fields] OR “ToF”[All Fields])

**Search Results:** 45 initial results, 8 results after exclusions

## Major Aortopulomary Collateral Arteries

**PubMed Search:** (("printing, three-dimensional"[MeSH Terms] OR ("printing"[All Fields] AND "three-dimensional"[All Fields]) OR "three-dimensional printing"[All Fields] OR ("3d"[All Fields] AND "printing"[All Fields]) OR "3d printing"[All Fields]) OR ("rapid"[All Fields] AND "prototyping"[All Fields]) OR ("additive"[All Fields] AND "manufacturing"[All Fields])) AND ("Major aortopulmonary"[All Fields] OR " Multiple aortopulmonary"[All Fields] OR ("MAPCA"[All Fields] OR " MAPCAs"[All Fields])

**Search Results:** 1 initial result, 0 results after exclusions

## Tricuspid Valve Disease and Ebstein’s Anomaly

**PubMed Search:** ((“printing, three-dimensional”[MeSH Terms] OR (“printing”[All Fields] AND “three-dimensional”[All Fields]) OR “three-dimensional printing”[All Fields] OR (“3d”[All Fields] AND “printing”[All Fields]) OR “3d printing”[All Fields]) OR ("rapid"[All Fields] AND "prototyping"[All Fields]) OR ("additive"[All Fields] AND "manufacturing"[All Fields])) AND ("Tricuspid Atresia"[MeSH Terms] OR "Ebstein Anomaly"[MeSH Terms] OR “Tricuspid Atresia”[All Fields] OR “Tricuspid Valve Atresia”[All Fields] OR “Tricuspid Valve Disease”[All Fields] OR “Ebstein”[All Fields] OR “Ebstein's”[All Fields])

**Search Results:** 11 initial result, 7 results after exclusions

## Right Ventricular Outflow Tract (RVOT) Obstruction and Pulmonary Artery Stenosis

**PubMed Search:** (("printing, three-dimensional"[MeSH Terms] OR ("printing"[All Fields] AND "three-dimensional"[All Fields]) OR "three-dimensional printing"[All Fields] OR ("3d"[All Fields] AND "printing"[All Fields]) OR "3d printing"[All Fields]) OR ("rapid"[All Fields] AND "prototyping"[All Fields]) OR ("additive"[All Fields] AND "manufacturing"[All Fields])) AND ("RVOT Obstruction"[All Fields] OR ("Right"[All Fields] AND "Ventricular"[All Fields] AND "Outflow"[All Fields] "Obstruction"[All Fields]) OR "Pulmonary Artery Stenosis"[All Fields] OR "Pulmonary Artery Stenoses"[All Fields] OR "RVOTO"[All Fields])

**Search Results:** 11 initial results, 2 results after exclusions

## Hypoplastic Left Heart Syndrome (HLHS)

**PubMed Search:** ((“printing, three-dimensional”[MeSH Terms] OR (“printing”[All Fields] AND “three-dimensional”[All Fields]) OR “three-dimensional printing”[All Fields] OR (“3d”[All Fields] AND “printing”[All Fields]) OR “3d printing”[All Fields]) OR ("rapid"[All Fields] AND "prototyping"[All Fields]) OR ("additive"[All Fields] AND "manufacturing"[All Fields])) AND (“Hypoplastic Left Heart Syndrome”[MESH Terms] OR “Hypoplastic Left Heart”[All Fields] OR “Hypoplastic Left Ventricle”[All Fields] OR “Left Heart Hypoplasia”[All Fields])

**Search Results:** 9 initial results, 2 results after exclusions

## Single Ventricle

**PubMed Search:** (("printing, three-dimensional"[MeSH Terms] OR ("printing"[All Fields] AND "three-dimensional"[All Fields]) OR "three-dimensional printing"[All Fields] OR ("3d"[All Fields] AND "printing"[All Fields]) OR "3d printing"[All Fields]) OR ("rapid"[All Fields] AND "prototyping"[All Fields]) OR ("additive"[All Fields] AND "manufacturing"[All Fields])) AND ("Univentricular Heart"[MESH Terms] OR "Univentricular Heart"[All Fields] OR "Single Ventricle"[All Fields]) OR (“Double Inlet Left Ventricle”[All Fields] OR “DILV”[All Fields]) OR (“Double Inlet Right Ventricle”[All Fields] OR “DIRV”[All Fields])

**Search Results:** 11 initial results, 0 results after exclusions

## Heterotaxy, Cardiac Anomaly

**PubMed Search:** (("printing, three-dimensional"[MeSH Terms] OR ("printing"[All Fields] AND "three-dimensional"[All Fields]) OR "three-dimensional printing"[All Fields] OR ("3d"[All Fields] AND "printing"[All Fields]) OR "3d printing"[All Fields]) OR ("rapid"[All Fields] AND "prototyping"[All Fields]) OR ("additive"[All Fields] AND "manufacturing"[All Fields])) AND (“heterotaxy”[All Fields] OR “heterotaxia”[All Fields])

**Search Results:** 3 initial results, 1 result after exclusions

## Levo-Corrected Transposition of the Great Arteries (L-TGA)

**PubMed Search:** (("printing, three-dimensional"[MeSH Terms] OR ("printing"[All Fields] AND "three-dimensional"[All Fields]) OR "three-dimensional printing"[All Fields] OR ("3d"[All Fields] AND "printing"[All Fields]) OR "3d printing"[All Fields]) OR ("rapid"[All Fields] AND "prototyping"[All Fields]) OR ("additive"[All Fields] AND "manufacturing"[All Fields])) AND ("Congenitally Corrected Transposition of the Great Arteries"[MESH Terms] OR "Congenitally Corrected"[All Fields] OR "LTGA"[All Fields] OR "L-TGA"[All Fields] OR "Levo-Transposition of the Great Arteries"[All Fields] OR "Levo-Transposition"[All Fields] OR "Levo Transposition"[All Fields])

**Search Results:** 5 initial results, 5 results after exclusions

## Dextro-Transposition of the Great Arteries (D-TGA)

**PubMed Search:** (("printing, three-dimensional"[MeSH Terms] OR ("printing"[All Fields] AND "three-dimensional"[All Fields]) OR "three-dimensional printing"[All Fields] OR ("3d"[All Fields] AND "printing"[All Fields]) OR "3d printing"[All Fields]) OR ("rapid"[All Fields] AND "prototyping"[All Fields]) OR ("additive"[All Fields] AND "manufacturing"[All Fields])) AND ("Transposition of Great Vessels"[MESH Terms] OR "DTGA"[All Fields] OR "D-TGA"[All Fields] OR "Dextro-Transposition"[All Fields] OR "Transposition of Great Vessels"[All Fields] OR "Transposition of Great Arteries"[All Fields])

**Search Results:** 24 initial results, 5 results after exclusions

## Double Outlet Right Ventricle (DORV)

**PubMed Search:** ((“printing, three-dimensional”[MeSH Terms] OR (“printing”[All Fields] AND “three-dimensional”[All Fields]) OR “three-dimensional printing”[All Fields] OR (“3d”[All Fields] AND “printing”[All Fields]) OR “3d printing”[All Fields]) OR ("rapid"[All Fields] AND "prototyping"[All Fields]) OR ("additive"[All Fields] AND "manufacturing"[All Fields])) AND (“Double Outlet Right Ventricle”[MESH Terms] OR “Double Outlet Right”[All Fields] OR “Double Outlet Right Ventricle”[All Fields] OR “DORV”[All Fields])

**Search Results:** 13 initial results, 25 results after exclusions

## Atrioventricular and/or Ventriculoarterial Discordance and (excluding Single Ventricle, TGA, and DORV)

**PubMed Search:** ((“printing, three-dimensional”[MeSH Terms] OR (“printing”[All Fields] AND “three-dimensional”[All Fields]) OR “three-dimensional printing”[All Fields] OR (“3d”[All Fields] AND “printing”[All Fields]) OR “3d printing”[All Fields]) OR ("rapid"[All Fields] AND "prototyping"[All Fields]) OR ("additive"[All Fields] AND "manufacturing"[All Fields])) AND ((“Double Outlet Left Ventricle”[All Fields] OR “DOLV”[All Fields]) OR (“criss-cross”[All Fields]) OR (“superior-inferior ventricles”[All Fields]) OR (“upstairs-downstairs”[All Fields]))

**Search Results:** 5 initial results, 3 results after exclusions
