# Supplementary material for: Clinical situations for which 3D printing is considered an appropriate representation or extension of data contained in a medical imaging examination: pediatric congenital heart disease conditions
Source: 3D Print Med. 2024 Jan 29;10:3. doi: 10.1186/s41205-023-00199-3 (PMC10823658; doi:10.1186/s41205-023-00199-3)
Supplement: Supplementary file 2 — Supplementary Material 2: Level of evidence for publications in the appropriateness document [file 41205_2023_199_MOESM2_ESM.docx]

# Appendix II: Level of Evidence Information

## Background

Grading of each included study with a strength of evidence assessment according to ACR Appropriateness Criteria Evidence Document. Studies were categorized as either primarily diagnostic (Dx), therapeutic (Tx), or both (Dx and Tx) along with a designation of observational, experimental, or review/other category. The review/other category is designated for studies that did not meet the definitions of the ACR Evidence Document for observational or experimental studies.

## Level of Evidence Information

| Ref | Disease | First Author | Title | Study Categories | Sample Size | Year | Category | Study Objective | Study Results |  |
| --- | --- | --- | --- | --- | --- | --- | --- | --- | --- | --- |
| 4 | ASD additional Complexity | So | Using multimaterial 3-dimensional printing for personalized planning of complex structural heart disease intervention | Review Other | 1 | 2017 | Category 4 | Authors 3D print a model of a atrial septal occluder device with partial prolapse through a secundum atrial septal defect. The model was used for pre-procedural planning prior to reintervention. | Simulated device closure was conducted on the model. The actual procedure was successfully performed, with excellent agreement with the simulation. | |
| 5 | ASD additional Complexity | Lee | Using a 3-Dimensional Printed Model to Plan Percutaneous Closure of an Unroofed Coronary Sinus | Review Other | 1 | 2021 | Category 4 | Authors 3D print a model for a patient with an unroofed coronary sinus atrial septal defect to aid in planning percutaneous device closure. | Bench testing of various percutaneous closure devices was performed using the 3D model. The first choice of device failed push-pull testing during cardiac catheterization. The second device was deployed successfully. | |
| 6 | ASD additional Complexity, PAPVR | Thakkar | Transcatheter closure of a sinus venosus atrial septal defect using 3D printing and image fusion guidance | Review Other | 1 | 2018 | Category 4 | A 3D model printed from CTA was used to simulate device deployment, demonstrating successful exclusion of the sinus venosus ASD with return of the RUPV to the left atrium | Case demonstrates comprehensive use of contemporary imaging for planning, simulation, patient consent, and procedural guidance for complex structural intervention in repair of sinus venosus ASD with PAPVR. | |
| 7 | ASD additional Complexity | Wang | Three-dimensional printing-guided percutaneous transcatheter closure of secundum atrial septal defect with rim deficiency: First-in-human series | Observational Tx | 6 | 2016 | Category 3 | Authors evaluate the use of 3-dimensional printing for the percutaneous transcatheter closure of a secundum atrial septal defect with a deficient rim less than 5 mm. | The final occluder size agreed with the occluder selected from simulation in 4 out of 5 cases. | |
| 8 | ASD additional Complexity | Luo | Three-dimensional printing model-guided percutaneous closure of atrial septal defect | Review Other | 1 | 2017 | Category 4 | Authors 3D print a model for a patient with an inferior sinus venous atrial septal defect to aid in planning percutaneous device closure. | Authors tried various sizes of ASD occluders on the model to completely cover the defect. The occluder size that was selected in bench testing of the model was successfully deployed with out residual shunting. | |
| 9 | ASD additional Complexity | Wang | Three-dimensional printing model for the postoperative follow-up of atrial septal defect | Review Other | 1 | 2016 | Category 4 | A 34 mm occluder device was used to close an inferior sinus venosus atrial septal defect. A month later the patient underwent multi-slice computed tomography (MSCT) that indicated no shift of the ASD occluder. A three-dimensional heart model was printed using the MSCT. | 3D printing can serve as an ideal tool for the visualization of ASD occluder and thus aid in the recognition of possible postoperative complications. | |
| 10 | ASD additional Complexity | Bartel | Three-dimensional printing for quality management in device closure of interatrial communications | Review Other | 1 | 2016 | Category 4 | Authors used a 3D model to evaluate the location of an atrial septal occluder device after deploment. | The virtual and tangible 3D models demonstrated the device to adequately fill the ASD and both countercluders to not interfere with venous inlets. | |
| 11 | ASD additional Complexity | Yan | Three-dimensional printing assisted transcatheter closure of atrial septal defect with deficient posterior-inferior rim | Observational Tx | 35 | 2018 | Category 3 | Authors use personalized heart models to plan percutaneous transcatheter closure in a series of patients with secundum atrial septal defects and deficient posterior-inferior rims. | Successful in vitro occlusion was achieved in 30 patients. The subsequent transcatheter closure was performed successfully in 29 patients, and the occluder-diameter was identical to that of in vitro occlusion. | |
| 12 | ASD additional Complexity | Chaowu | Three-dimensional printing as an aid in transcatheter closure of secundum atrial septal defect with rim deficiency: in vitro trial occlusion based on a personalized heart model | Review Other | 1 | 2016 | Category 4 | Authors use a personalized heart model for planning percutaneous closure of a secundum atrial septal defect with a deficient posterior-inferior rim. | Position and morphology of the device were in good agreement between the pre-intervention trial occlusion and post-intervention occlusion. | |
| 13 | ASD | Faganello | Three dimensional printing of an atrial septal defect: Is it multimodality imaging? | Review Other | 1 | 2016 | Category 4 | Authors print a limited section of atrial septum from 3D transesophageal echocardiography. | Size of the atrial septal defect in the 3D model agrees with measurements from transesophageal echocardiography. | |
| 14 | ASD additional Complexity | Li | Personalized Three-Dimensional Printing and Echoguided Procedure Facilitate Single Device Closure for Multiple Atrial Septal Defects | Experimental Tx | 62 | 2020 | Category 3 | Sixty-two patients with multiple ASDs were retrospectively analyzed. Thirty of these patients underwent TTE-guided closure (3D printing and TTE group) after a simulation of occlusion in 3D printing models. The remaining 32 patients underwent ASD closure under fluoroscopic guidance (conventional group). | Successful transcatheter closure with a single device was achieved in 26 patients in the 3D printing and TTE group and 27 patients in the conventional group. The 3D printing and TTE group had lower frequency of occluder replacement. | |
| 15 | ASD additional Complexity | Yan | Off-label use of duct occluder in transcatheter closure of secundum atrial septal defect with no rim to right pulmonary vein | Observational Tx | 7 | 2019 | Category 3 | Seven consecutive patients with an atrial septal defect with no right pulmonary vein rim were referred for attempted transcatheter closure, and a personalized heart model was produced from multislice computed tomography images. With the elastic heart model, in vitro trial occlusion was performed with a duct occluder for preoperative evaluation. | In vitro trial occlusion was achieved in all patients, and subsequent in vivo transcatheter closure was performed successfully with the duct occluder of the same size. | |
| 16 | ASD additional Complexity, PAPVR | Velasco Forte | Interventional correction of sinus venosus atrial septal defect and partial anomalous pulmonary venous drainage: procedural planning using 3D printed models | Review Other | 3 | 2017 | Category 4 | Patient-specific 3-dimensional printing and in vitro simulation was used to plan interventional catheterization treatment in 3 adult patients with sinus venosus atrial septal defects and partial anomalous pulmonary venous drainage. | Simulation of the procedure gave the authors confidence that the pulmonary veins would remain patent before the clinical catheterization. | |
| 17 | ASD additional Complexity, PAPVR | Butera | Holographic augmented reality and 3D printing for advanced planning of sinus venosus ASD/partial anomalous pulmonary venous return percutaneous management | Review Other | 1 | 2019 | Category 4 | 3D printing and a holographic augmented reality were used to plan interventional catheterization treatment in a patient with a sinus venosus atrial septal defect and partial anomalous pulmonary venous drainage. | All tests were successful, and the patient underwent implantation of a custom-made covered stent in the SVC-to-right atrium junction to exclude the SVASD while directing across it the anomalous pulmonary venous return toward the left atrium. | |
| 18 | VSD additional Complexity | Mendez | Apical Muscular Ventricular Septal Defects: Surgical Strategy Using Three-Dimensional Printed Model | Review Other | 1 | 2018 | Category 4 | A 3D model was used to plan surgical patch closure of multiple apical ventricular septal defects. | The 3D model helped identifying the total number of VSD, their origins and exits as well as the minimum number of septal bands to resect, the optimal patch size and morphology to completely close all VSDs without reducing significantly the RV cavity. | |
| 19 | VSD additional Complexity | Bhatla | Utility and Scope of Rapid Prototyping in Patients with Complex Muscular Ventricular Septal Defects or Double-Outlet Right Ventricle: Does it Alter Management Decisions? | Review Other | 3 | 2019 | Category 4 | Virtual and physical 3D models were generated from CT for 3 patients with complex muscular ventricular septal defect. The models were used to guide management decisions. | The authors believe these models improve understanding of the complex anatomical spatial relationships in these defects and provide additional insight for pre/intra-interventional management and surgical planning. | |
| 20 | VSD | Sodian | Stereolithographic Models for Surgical Planning in Congenital Heart Surgery | Review Other | 1 | 2007 | Category 4 | A stereolithographic model was created for a patient with a subpulmonary ventricular septal defect. | The model showed the dimensions of the defect and surrounding anatomic structures. | |
| 21 | VSD | Liang | Comparison of blood pool and myocardial 3D printing in the diagnosis of types of congenital heart disease | Review Other | 1 | 2022 | Category 4 | Blood pool and myocardial models were created for a patient with a ventricular septal defect. The models were evaluated by groups of experts and students. | Myocardical models of the ventricular septal defect were preferred. | |
| 22 | VSD additional Complexity | Ghosh | Clinical 3D modeling to guide pediatric cardiothoracic surgery and intervention using 3D printed anatomic models, computer aided design and virtual reality | Review Other | 11 | 2022 | Category 4 | The authors review the growth and development of a clinical 3D modeling service to inform procedural planning within a high-volume pediatric heart center. | Over a three year period eleven 3D models were created for the indication of multiple ventricular septal defects. | |
| 23 | VSD additional Complexity | Bhatla | Altering management decisions with gained anatomical insight from a 3D printed model of a complex ventricular septal defect | Review Other | 1 | 2017 | Category 4 | A 3D model was created for an infant with a comple tunnel-like ventricular septal defect. | The understanding provided by the three-dimensional model helped obtain additional focused echocardiographic data to characterize the high-pressure restriction across the defect. | |
| 24 | AV Canal | Veronese | Three‑dimensional printing of the fetal heart with complete atrioventricular septal defect based on ultrasound data | Review Other | 1 | 2020 | Category 4 | Authors sought to explore the possibility of reconstructing and printing a 3D model of the fetal heart affected by complete atrioventricular septal defect (cAVSD), based on prenatal ultrasound images. | Authors present a case study of a 3D printed heart model of the fetus with cAVSD. | |
| 25 | AP Window | Moore | Three-dimensional printing in surgical planning A case of aortopulmonary window with interrupted aortic arch | Review Other | 1 | 2018 | Category 4 | Authors sought to better understanding and conceptualize complex congenital heart defects using three-dimensional (3D) printing for rare defects. | Authors utilized 3D printing to delineate the exact cardiac anatomy of a neonate with an aortopulmonary window associated with interrupted aortic arch to devise a novel approach to the repair. | |
| 26 | AP Window | Balegadde | A case of asymptomatic large aortopulmonary window in an adult: Role of cardiac CT, CMRI, and 3D printing technology | Review Other | 1 | 2018 | Category 4 | Authors report an unusual case of a 32-year-old female with a large, unrepaired AP Window causing severe pulmonary hypertension. | The patient's anatomy was not ammenable to an interventional approach after review of the 3D print. | |
| 27 | IAAwLVOTO, Tetralogy of Fallot, MAPCAs, HLHS | Hoashia | Utility of a super-flexible three-dimensional printed heart model in congenital heart surgery | Experimental Tx | 20 | 2018 | Category 3 | The objective of this study was to assess the utility of our original 3D heart models of congenital heart disease for the benefit of inexperienced consultant surgeons. | 3D printed heart models seem useful for understanding the relationship between intraventricular communications and great vessels and simulations for the creation of intracardiac pathways. | |
| 28 | Truncus Arteriosus | Biglino | The Perception of a Three-Dimensional-Printed Heart Model from the Perspective of Different Stakeholders: A Complex Case of Truncus Arteriosus | Review Other | 1 | 2017 | Category 4 | The case of an 11-year-old male patient with truncus arteriosus is presented. Prior to the surgery and to facilitate communication with the patient’s parents, a three-dimensional (3D) model of his heart and main vessels was created. | Feedback was collected from different stakeholders and presented in a qualitative format. | |
| 29 | PAPVR, TAPVR | Xu | Utility of three-dimensional printing in preoperative planning for children with anomalous pulmonary venous connection: a single center experience | Observational Tx | 17 | 2019 | Category 3 | This study sought to assess the application of three-dimensional (3D) printing in preoperative planning for anomalous pulmonary venous connection (APVC). | 3D heart models accurately demonstrated the malformations, which were all confirmed consistent with surgery findings. The authors state that 3D printing is beneficial for preoperative planning and post-surgery follow-up in APVC. | |
| 30 | Tetralogy of Fallot | Lodzinki | Three-dimensional print facilitated ventricular tachycardia ablation in patient with corrected congenital heart disease | Review Other | 1 | 2017 | Category 4 | Authors present a case study of a 52 year old female with post-repair anatomy. A 3D print was utilizaed in planning for an ablation. | A 3D print as produced and used to plan for an ablation. He result was a permanent termination of the tachycardia. | |
| 31 | Tetralogy of Fallot | Ma | Clinical application of three-dimensional reconstruction and rapid prototyping technology of multislice spiral computed tomography angiography for the repair of ventricular septal defect of tetralogy of Fallot | Observational Tx | 35 | 2015 | Category 3 | Three-dimensional reconstruction and 3D printing was applied to prepare physical models of the heart and ventricular septal defects of tetralogy of Fallot (ToF) patients in order to explore their applications in the diagnosis and treatment of this complex heart disease. | For all 35 patients, solid models of the heart shape and ventricular septal defect were created using 3D reconstruction and 3D printing. All surgeries were completed according to the preoperative surgical planning and were uneventful. | |
| 32 | Tetralogy of Fallot | Averkin | 3D-printing in preoperative planning in neonates with complex congenital heart defects | Review Other | 1 | 2020 | Category 4 | The study aimed to demonstrate the authors experience in using 3D printing for preoperative management planning of the newborn with TOF, combined with APVS. | A 3D print was developed for a newborn male for surgical planning. The authors demonstrate the application of 3 D model technology in a patient with complex cardiac defects, who was treated successfully, thanks to a detailed assessment of the existing defect. | |
| 33 | Tetralogy of Fallot | Deferm | 3D-Printing in Congenital Cardiology: From Flatland to Spaceland | Review Other | 1 | 2016 | Category 4 | The authors printed pre‑ and postoperative 3D‑models of a complex congenital heart defect. The authors attempt to show that the models hold value in preoperative planning, postoperative evaluation of a complex procedure, communication with the patient, and education of trainees. | A multidisciplinary team used a printed 3D model to discuss the preoperative procedure in detail and afterward with the patient. A post-procedure 3D was also developed to understand patency of conduit. | |
| 34 | MAPCAs | Qiu | A Novel 3D Visualized Operative Procedure in the Single-Stage Complete Repair With Unifocalization of Pulmonary Atresia With Ventricular Septal Defect and Major Aortopulmonary Collateral Arteries | Experimental Tx | 20 | 2022 | Category 3 | The purpose of this study is to summarize the authors' experience of a novel three-dimensional (3D) visualized operative procedure in the single-stage complete repair with unifocalization and to clarify the benefits it may bring. | The novel 3D visualized operative procedure may help improve the performance of the single-stage complete repair with the midline unifocalization of PA/VSD/MAPCAs and shorten the dissecting time of the MAPCAs. | |
| 35 | MAPCAs | Cen | Three-Dimensional Printing, Virtual Reality and Mixed Reality for Pulmonary Atresia: Early Surgical Outcomes Evaluation | Observational Tx | 5 | 2021 | Category 4 | Authors seek to evaluate the benefit of 3D printing and holographic visualisation technology on surgical outcomes. This study retrospectively analysed five selected PA/VSD/MAPCA patients who underwent single-stage unifocalisation through the midline. | The preoperative application of a 3D-printed heart model with VR or MR helped in aligning the surgical field. These technologies improved the understanding of complicated cardiac anatomy and achieved acceptable surgical outcomes as guiding surgical planning. | |
| 36 | MAPCAs | Jivanji | Novel use of a 3D printed heart model to guide simultaneous percutaneous repair of severe pulmonary regurgitation and right ventricular outflow tract aneurysm | Review/Other | 1 | 2019 | Category 4 | The authors describe percutaneous repair of severe pulmonary regurgitation and a right ventricular outflow tract pseudoaneurysm in a 19-year-old patient after repair of pulmonary atresia, ventricular septal defect, and major aortopulmonary collaterals. | The encouraging findings from the simulation on the 3D print allowed the authors to plan the complex procedure effectively with a successful outcome and avoidance of surgery. | |
| 37 | RVOT and PS, MAPCAs | Parimi | Feasibility and Validity of Printing 3D Heart Models from Rotational Angiography | Observational Tx | 5 | 2018 | Category 2 | The objective of this case series is to evaluate and validate rotational angiography based 3D printed models for patients with complex congenital heart disease. | Rotational angiography can generate highly accurate 3D models in congenital heart disease, including in small vascular structures. These models can be extremely useful in patient evaluation and management. | |
| 38 | MAPCAs | Ngan | The rapid prototyping of anatomic models in pulmonary atresia | Observational Combined | 6 | 2006 | Category 3 | The goal of this study was to assess the utility and accuracy of 3D printed anatomic models for surgical planning in patients with pulmonary atresia with ventricular septal defect and major aortopulmonary collateral arteries. | Anatomic models are an intuitive means of communicating complex imaging data, such as the pulmonary vascular tree, which can be referenced intraoperatively. | |
| 39 | HLHS | Kiraly | Three-dimensional printed prototypes refine the anatomy of post-modified Norwood-1 complex aortic arch obstruction and allow presurgical simulation of the repair | Experimental Tx | 1 | 2016 | Category 3 | The objective of this case study is to demonstrate how a printed 3D model depicted the precise representation of the spatial relationships of anatomical structures in an infant with complex neo-aortic arch obstruction and allowed simulation of the repair. | The operation was performed in accordance with preoperative simulation and the knowledge gained from the models fundamentally contributed to successful outcome and improved patient safety. | |
| 40 | Heterotaxy | Bettencourt | Utility of Three-Dimensional Printed Model in Biventricular Repair of Complex Congenital Cardiac Defects: Case Report and Review of Literature | Review/Other | 1 | 2022 | Category 4 | To report the use of a 3D printed cardiac model to plan a unique biventricular repair using a Warden-like procedure in a patient with heterotaxy syndrome and complex cardiac anatomy. | This case highlights the use of a 3D-printed model in helping plan a unique surgical approach to achieve a successful biventricular repair in a patient with heterotaxy syndrome. 3D printing is proving to be an integral tool in understanding the anatomical complexity, planning the surgical approach, and achieving successful outcomes for patients with complex congenital heart defect | |
| 41 | DORV, LTGA | Kappanayil | Three-dimensional-printed cardiac prototypes aid surgical decision-making and preoperative planning in selected cases of complex congenital heart diseases: Early experience and proof of concept in a resource-limited environment | Experimental Tx | 5 | 2017 | Category 3 | The objective of this study was to explore the feasibility and impact of using patient-specific 3D-printed cardiac prototypes derived from high-resolution medical imaging data (cardiac magnetic resonance imaging/computed tomography [MRI/CT]) on surgical decision-making and preoperative planning in selected cases of complex congenital heart diseases (CHDs) | 3D-printed cardiac prototypes can radically assist decision-making, planning, and safe execution of complex congenital heart surgery by improving understanding of 3D anatomy and allowing anticipation of technical challenges. | |
| 42 | L-TGA | Gosnell | Integration of Computed Tomography and Three-Dimensional Echocardiography for Hybrid Three-Dimensional Printing in Congenital Heart Disease | Review/Other | 1 | 2016 | Category 4 | Case report describing feasibility of hybrid 3D printing from two imaging modalities in a patient with congenitally corrected transposition of the great arteries (L-TGA) | Hybrid 3D printing may be useful as an additional tool for cardiologists and cardiothoracic surgeons in planning interventions in children and adults with CHD. | |
| 43 | L-TGA | Cho | Hemodynamically balanced congenitally corrected transposition of the great arteries with a large ventricular septal defect, and subvalvular pulmonic stenosis: A case report | Review/Other | 1 | 2019 | Category 4 | To report an adult case of unoperated congenitally corrected transposition of the great arteries with a large ventricular septal defect and probable pulmonary arterial hypertension | A detailed anatomical understanding based on transthoracic echocardiography, cardiac computed tomography, and three-dimensional printing can justify a decision to not operate in cases of congenitally corrected transposition of the great arteries with hemodynamically balanced pulmonary stenosis and a ventricular septal defect | |
| 44 | L-TGA | Sahayaraj | 3D Printing to Model Surgical Repair of Complex Congenitally Corrected Transposition of the Great Arteries | Review/Other | 1 | 2019 | Catgeory 4 | Toreport the use of three-dimensional (3D) modeling to plan surgery for physiologic repair of congenitally corrected transposition of the great arteries with pulmonary atresia, dextrocardia, and complex intra cardiac anatomy | The physiologic repair of a complex lesion has been achieved using a composite conduit consisting of three different types of grafts, taking the advantage of 3D printing and modeling in planning of the repair. Use of newer technology, such as 3D printing, makes decision-making and surgical planning simpler, in complex cardiac defects | |
| 45 | D-TGA | Xu | Patient-specific three-dimensional printed heart models benefit preoperative planning for complex congenital heart disease | Experimental Tx | 15 | 2019 | Category 3 | To investigate the roles of three-dimensional printed patient-specific heart models in the presurgical planning for complex congenital heart disease. | 3D printed heart models are beneficial and promising in preoperative planning for complex congenital heart diseases | |
| 46 | D-TGA | Miller | 3D printing for preoperative planning and surgical simulation of ventricular assist device implantation in a failing systemic right ventricle | Review/Other | 1 | 2020 | Category 4 | To describe the application of 3D printing for surgical simulation of VAD placement in a complex ACHD patient with systemic right ventricle. | Successful surgical simulation of VAD placement in the systemic right ventricle of a patient with complex congenital heart disease. | |
| 47 | D-TGA | Olivieri | 3D heart model guides complex stent angioplasty of pulmonary venous baffle obstruction in a Mustard repair of D-TGA | Review/Other | 1 | 2014 | Category 4 | Case report detailing utilization of non-invasive imaging to create anatomically correct 3D printed models of complex cardiac anatomy for surgical planning. | This case demonstrates how a printed model of structural heart disease can help plan the interventional approach, and inform the selection of the appropriate catheter course and device. | |
| 48 | D-TGA | Poterucha | Percutaneous Pulmonary Valve Implantation in a Native Outflow Tract | Review/Other | 1 | 2014 | Category 4 | Describe utilization of DynaCT and 3D printing to guide percutaneous pulmonary valve placement. | This is the first reported case of percutaneous pulmonary valve implantation into a native RVOT using 3D rotational angiography for guidance of stent and valve implantation. Three-dimensional printed models provide a novel and valuable tool for patient and trainee education. | |
| 49 | DORV | Farooqi | Use of a Three Dimensional Printed Cardiac Model to Assess Suitability for Biventricular Repair | Review/Other | 1 | 2016 | Category 4 | Authors present the case of a seven-year-old boy with double outlet right ventricle who underwent a bidirectional Glenn anastomosis. Authors used a 3D cardiac model to assess his suitability for a biventricular repair | This case demonstrates application of this technology in a patient with a complex cardiac defect who was a candidate to transition from a single-ventricle palliation to a two-ventricle repair. Authors were able to clearly visualize a realistic potential baffle pathway in 3D which would allow septation of the ventricles. | |
| 50 | DORV | Shearn | Use of 3D Models in the Surgical Decision-Making Process in a Case of Double-Outlet Right Ventricle With Multiple Ventricular Septal Defects | Review/Other | 1 | 2019 | Category 4 | Authors report here the use of both rigid and flexible 3D models of a complex CHD case to aid surgical planning. | Given the high complexity of this particular case and the unique anatomy, the input of a 3D model was enormously helpful, not only for the understanding of its particular disease but also for planning a biventricular repair over a univentricular repair and the improvement of the surgical technique. | |
| 51 | DORV | Farooqi | Use of 3-dimensional printing to demonstrate complex intracardiac relationships in double-outlet right ventricle for surgical planning | Review/Other |  | 2015 | Category 4 | To describe the utility of 3d modeling and printing in a case with complex intracardiac anatomy. | The intricate complexity of this patient's anatomy demonstrates the optimal setting in which 3D intracardiac modeling and printing can offer a transition from virtual to real spatial modeling. | |
| 52 | DORV | Zhao | Three-dimensional printing enhances preparation for repair of double outlet right ventricular surgery | Experimental Tx | 25 | 2018 | Category 1 | This study was undertaken to assess the role of 3D printed models in planning pre-operative strategies for the surgical repair of DORV. | Patients in the 3Dprinting group had shorter aortic cross-clamp time (102.88 vs 127.76 min, P=0.094) and cardiopulmonary bypass time (151.63 vs 184.24 min; P=0.152) than patients in the control group. Patients with 3D printed models had significantly lower mechanical ventilation time (56.43 vs 96.76h, P=0.040) and significantly shorter intensive care unit time (99.04 vs 166.94h, P=0.008) than patients in the control group. | |
| 53 | DORV | Perens | Three-Dimensional Congenital Heart Models Created With Free Software and a Desktop Printer: Assessment of Accuracy, Technical Aspects, and Clinical Use | Review/Other | 6 | 2020 | Category 4 | To report the initial results for feasibility and clinical value in 3D printed models of five pediatric patients (3-6 months) and one adult with complex CHD. | Accurate 3D printed models of complex, pediatric CHD may be created from high-resolution volumetric MRI and CT studies using free online software and printed with an inexpensive desktop printer. This low-cost methodology would allow ready access for more pediatric CHD programs to create 3D models for preprocedural surgical planning and education. | |
| 54 | DORV | Bhatla | Surgical planning for a complex double-outlet right ventricle using 3D printing | Review/Other | 1 | 2017 | Category 4 | To describe the utilization of 3D printed models for surgical decision making in a patient with DORV. | The decision to obtain a three-dimensional printed model helped for better understanding of the anatomy, with the additional advantage of surgical simulation in planning the surgical approach and type of surgical repair. | |
| 55 | DORV | Garekar | Clinical Application and Multidisciplinary Assessment of Three Dimensional Printing in Double Outlet Right Ventricle With Remote Ventricular Septal Defect | Review/Other | 1 | 2016 | Category 4 | To present a case series of 5 patients with DORV. In this cohort the authors used a novel scoring system to compare the assessment of 3D printed heart model findings with information obtained from echocardiography, CT, or cardiac MRI and with details of the surgeon’s intraoperative direct observations | The 3D printed models scored higher than conventional imaging, with respect to most aspects of the surface spatial orientation and intracardiac anatomy. The models are a useful adjunct in preoperative assessment of complex DORV. | |
| 56 | DORV | Smith | The role of 3D printing in preoperative planning for heart transplantation in complex congenital heart disease | Review/Other | 1 | 2017 | Category 4 | The study attempts to demonstrate the benefit of 3D printing to enhance preoperative planning in complex congenital heart disease undergoing heart transplantation. | The authors demonstrated the pivotal role played by 3D printing in advancing spatial comprehension of complex aberrant anatomy. | |
| 57 | DOLV | Hadeed | Three-dimensional printing of a complex CHD to plan surgical repair | Review/Other | 1 | 2016 | Category 4 | This case demonstrates how this new innovative technology allows better understanding of the anatomy in complex CHDs and permits to better plan the surgical repair | The use of three-dimensional printing model removes the potential for misinterpretation that may occur between the presentation of images in two dimensions and the mental reconstruction and provides a better definition of intra-cardiac spatial relationship | |
| 58 | Atrioventricular and/or Ventriculoarterial Discordance | Seckeler | Transjugular Transseptal Approach for Left Ventricular Pacing Lead in an Adult With Criss-Cross Heart | Review/Other | 1 | 2019 | Category 4 | Case study illustrates the utility of 3D printing for a patient with criss-cross atrioventricular connections (focusing on left ventricular pacing lead placement). | The patient's LVEF improved. This case highlights the anatomic complexities, the need for a unique approach, and the role of 3D modeling. | |
| 59 | Tetralogy of Fallot | Chamberlain | Pre-procedural Three-dimensional Planning Aids in Transcatheter Ductal Stent Placement: A Single-Center Experience | Observational Tx | 12 | 2020 | Category 4 | The manuscript describes the use of three-dimensional(3D) patent ductus arteriosus(PDA) modeling to better define ductal anatomy to improve pre-procedural planning for ductal stent placement. | 3D modeling prior to ductal stent placement for ductal dependent pulmonary blood flow is useful in procedural planning, specifically for eligibility, access approach, and accurate ductal measurements.  *The publication was added by the voting group, not by the structured query. | |
