# Supplementary material for: Clinical situations for which 3D printing is considered an appropriate representation or extension of data contained in a medical imaging examination: pediatric congenital heart disease conditions
Source: 3D Print Med. 2024 Jan 29;10:3. doi: 10.1186/s41205-023-00199-3 (PMC10823658; doi:10.1186/s41205-023-00199-3)
Supplement: Supplementary file 3 — Supplementary Material 3: Publications of note related to 3D technologies and congenital heart disease, but not meeting eligibility criteria for inclusion in the appropriateness rating [file 41205_2023_199_MOESM3_ESM.docx]

# Appendix III: Publications of Note

## Background

Many additional, tangentially-related publications were included in the initial results from the structured searches; however, as these manuscripts had no direct patient care impact, they were excluded from being used in setting appropriateness ratings. Table 2 covers excluded manuscripts associated to Feasibility/Accuracy/Proof-of-Concept, Research or extended reality (XR), or Education/Training content.

## Clinical Scenarios:

Table 1: Manuscripts excluded from the results for appropriateness ratings are presented. The supplemental content is segmented by classification: Feasibility/Accuracy/Proof-of-Concept, Research or XR, and Education/Training. References are included in the Reference List.

| **Scenario** | **Classification of publication** | **References** |
| --- | --- | --- |
| Atrial Septal Defect | Feasibility/Accuracy/Proof-of-Concept | [1–3] |
|  | Research or XR | [4,5] |
|  | Review Paper | [6] |
|  | Education/Training | [7–10] |
| Ventricular Septal Defect | Feasibility/Accuracy/Proof-of-Concept | [11–14] |
|  | Research or XR | [15–17] |
|  | Education/Training | [8,10] |
| Atrioventricular Canal | Education/Training | [18] |
| ToF | Feasibility/Accuracy/Proof-of-Concept | [11,19–23] |
|  | Education/Training | [7,8,24–27] |
| HLHS | Feasibility/Accuracy/Proof-of-Concept | [28] |
|  | Research or XR | [29,30] |
|  | Education/Training | [7,18,31] |
| Mitral Atresia | Feasibility/Accuracy/Proof-of-Concept | [32] |
|  | Research or XR | [33,34] |
| Single Ventricle (other) | Research or XR | [35–38] |
| D TGA | Feasibility/Accuracy/Proof-of-Concept | [1] |
|  | Education/Training | [39] |
| DORV | Feasibility/Accuracy/Proof-of-Concept | [11,40] |
|  | Research or XR | [41–45] |
|  | Review Paper(s) | [6,46] |
|  | Education/Training | [47–49] |

Appendix III References:

1. Veronese P, Bertelli F, Cattapan C, Andolfatto M, Gervasi MT, Vida VL. Three-dimensional printing of the fetal heart with complete atrioventricular septal defect based on ultrasound data. Kardiol Pol. 2020;78:930–1.

2. Li P, Fang F, Qiu X, Xu N, Wang Y, Ouyang W-B, et al. Personalized Three-Dimensional Printing and Echoguided Procedure Facilitate Single Device Closure for Multiple Atrial Septal Defects. J Interv Cardiol. 2020;2020:1751025.

3. He L, Cheng G-S, Du Y-J, Zhang Y-S. Feasibility of Device Closure for Multiple Atrial Septal Defects With an Inferior Sinus Venosus Defect: Procedural Planning Using Three-Dimensional Printed Models. Heart Lung Circ. 2020;29:914–20.

4. Lin C, Liu L, Liu Y, Leng J. Recent developments in next-generation occlusion devices. Acta Biomater. 2021;128:100–19.

5. Wang G, Xi L, Li H, Wang Y, Wu C, Pan Z, et al. Experience in the Treatment of Pentalogy of Cantrell with Artificial Materials in a Single Clinical Center. Eur J Pediatr Surg. 2022;32:50–5.

6. Anwar S, Singh GK, Miller J, Sharma M, Manning P, Billadello JJ, et al. 3D Printing is a Transformative Technology in Congenital Heart Disease. JACC Basic Transl Sci. 2018;3:294–312.

7. Smerling J, Marboe CC, Lefkowitch JH, Pavlicova M, Bacha E, Einstein AJ, et al. Utility of 3D Printed Cardiac Models for Medical Student Education in Congenital Heart Disease: Across a Spectrum of Disease Severity. Pediatr Cardiol. 2019;40:1258–65.

8. Karsenty C, Guitarte A, Dulac Y, Briot J, Hascoet S, Vincent R, et al. The usefulness of 3D printed heart models for medical student education in congenital heart disease. BMC Med Educ. 2021;21:480.

9. Morais P, Tavares JMRS, Queirós S, Veloso F, D’hooge J, Vilaça JL. Development of a patient-specific atrial phantom model for planning and training of inter-atrial interventions. Med Phys. 2017;44:5638–49.

10. Hopfner C, Jakob A, Tengler A, Grab M, Thierfelder N, Brunner B, et al. Design and 3D printing of variant pediatric heart models for training based on a single patient scan. 3D Print Med. 2021;7:25.

11. Lee S, Squelch A, Sun Z. Quantitative Assessment of 3D Printed Model Accuracy in Delineating Congenital Heart Disease. Biomolecules. 2021;11:270.

12. Mattus MS, Ralph TB, Keller SMP, Waltz AL, Bramlet MT. Creation of Patient-Specific Silicone Cardiac Models with Applications in Pre-surgical Plans and Hands-on Training. J Vis Exp. 2022;

13. Hadeed K, Guitarte A, Briot J, Dulac Y, Alacoque X, Acar P, et al. Feasibility and accuracy of printed models of complex cardiac defects in small infants from cardiac computed tomography. Pediatr Radiol. 2021;

14. Olivieri LJ, Krieger A, Loke Y-H, Nath DS, Kim PCW, Sable CA. Three-dimensional printing of intracardiac defects from three-dimensional echocardiographic images: feasibility and relative accuracy. J Am Soc Echocardiogr. 2015;28:392–7.

15. Xu C, Liu Y, Zhai M, Jin P, Li L, Ma Y, et al. Transcatheter Closure of a Paravalvular Leak Guided by Transesophageal Echocardiography and Three-Dimensional Printing. Front Cardiovasc Med. 2022;9:750896.

16. Ghosh RM, Mascio CE, Rome JJ, Jolley MA, Whitehead KK. Use of Virtual Reality for Hybrid Closure of Multiple Ventricular Septal Defects. JACC Case Rep. 2021;3:1579–83.

17. Deng X, He S, Huang P, Luo J, Yang G, Zhou B, et al. A three-dimensional printed model in preoperative consent for ventricular septal defect repair. J Cardiothorac Surg. 2021;16:229.

18. Ilina A, Lasso A, Jolley MA, Wohler B, Nguyen A, Scanlan A, et al. Patient-specific pediatric silicone heart valve models based on 3D ultrasound. Proc SPIE Int Soc Opt Eng. 2017;10135.

19. Liang J, Zhao X, Pan G, Zhang G, Zhao D, Xu J, et al. Comparison of blood pool and myocardial 3D printing in the diagnosis of types of congenital heart disease. Sci Rep. 2022;12:7136.

20. Parimi M, Buelter J, Thanugundla V, Condoor S, Parkar N, Danon S, et al. Feasibility and Validity of Printing 3D Heart Models from Rotational Angiography. Pediatr Cardiol. 2018;39:653–8.

21. Seckeler MD, Boe BA, Barber BJ, Berman DP, Armstrong AK. Use of rotational angiography in congenital cardiac catheterisations to generate three-dimensional-printed models. Cardiol Young. 2021;1–5.

22. Tomov ML, Cetnar A, Do K, Bauser‐Heaton H, Serpooshan V. Patient‐Specific 3‐Dimensional–Bioprinted Model for In Vitro Analysis and Treatment Planning of Pulmonary Artery Atresia in Tetralogy of Fallot and Major Aortopulmonary Collateral Arteries. J Am Heart Assoc. 2019;8:e014490.

23. Schievano S, Migliavacca F, Coats L, Khambadkone S, Carminati M, Wilson N, et al. Percutaneous Pulmonary Valve Implantation Based on Rapid Prototyping of Right Ventricular Outflow Tract and Pulmonary Trunk from MR Data. Radiology. 2007;242:490–7.

24. Nam JG, Lee W, Jeong B, Park EA, Lim JY, Kwak Y, et al. Three-Dimensional Printing of Congenital Heart Disease Models for Cardiac Surgery Simulation: Evaluation of Surgical Skill Improvement among Inexperienced Cardiothoracic Surgeons. Korean J Radiol. 2021;22:706–13.

25. Hussein N, Honjo O, Haller C, Hickey E, Coles JG, Williams WG, et al. Hands-On Surgical Simulation in Congenital Heart Surgery: Literature Review and Future Perspective. Seminars in Thoracic and Cardiovascular Surgery. 2020;32:98–105.

26. White SC, Sedler J, Jones TW, Seckeler M. Utility of three-dimensional models in resident education on simple and complex intracardiac congenital heart defects. Congenital Heart Disease. 2018;13:1045–9.

27. Loke Y-H, Harahsheh AS, Krieger A, Olivieri LJ. Usage of 3D models of tetralogy of Fallot for medical education: impact on learning congenital heart disease. BMC Med Educ. 2017;17:54.

28. Chen SA, Ong CS, Malguria N, Vricella LA, Garcia JR, Hibino N. Digital Design and 3D Printing of Aortic Arch Reconstruction in HLHS for Surgical Simulation and Training. World J Pediatr Congenit Heart Surg. 2018;9:454–8.

29. Sandrini C, Lombardi C, Shearn AIU, Ordonez MV, Caputo M, Presti F, et al. Three-Dimensional Printing of Fetal Models of Congenital Heart Disease Derived From Microfocus Computed Tomography: A Case Series. Front Pediatr. 2019;7:567.

30. Ruedinger KL, Zhou H, Trampe B, Heiser T, Srinivasan S, Iruretagoyena JI, et al. Modeling Fetal Cardiac Anomalies From Prenatal Echocardiography With 3-Dimensional Printing and 4-Dimensional Flow Magnetic Resonance Imaging. Circ Cardiovasc Imaging. 2018;11:e007705.

31. Hussein N, Honjo O, Barron DJ, Haller C, Coles JG, Van Arsdell G, et al. Assessment tool validation and technical skill improvement in the simulation of the Norwood operation using three-dimensional-printed heart models. Eur J Cardiothorac Surg. 2020;ezaa321.

32. Owais K, Pal A, Matyal R, Montealegre-Gallegos M, Khabbaz KR, Maslow A, et al. Three-dimensional printing of the mitral annulus using echocardiographic data: science fiction or in the operating room next door? J Cardiothorac Vasc Anesth. 2014;28:1393–6.

33. Wiener PC, Darwish A, Friend E, Kadem L, Pressman GS. Energy loss associated with in-vitro modeling of mitral annular calcification. PLoS One. 2021;16:e0246701.

34. Wang DD, Eng MH, Greenbaum AB, Myers E, Forbes M, Karabon P, et al. Validating a prediction modeling tool for left ventricular outflow tract (LVOT) obstruction after transcatheter mitral valve replacement (TMVR). Catheter Cardiovasc Interv. 2018;92:379–87.

35. McHugo S, Nolke L, Delassus P, MacCarthy E, Morris L, McMahon CJ. An in-vitro evaluation of the flow haemodynamic performance of Gore-Tex extracardiac conduits for univentricular circulation. J Cardiothorac Surg. 2020;15:235.

36. Cheng AL, Wee CP, Pahlevan NM, Wood JC. A 4D flow MRI evaluation of the impact of shear-dependent fluid viscosity on in vitro Fontan circulation flow. Am J Physiol Heart Circ Physiol. 2019;317:H1243–53.

37. Granegger M, Thamsen B, Hubmann EJ, Choi Y, Beck D, Valsangiacomo Buechel E, et al. A long-term mechanical cavopulmonary support device for patients with Fontan circulation. Med Eng Phys. 2019;70:9–18.

38. Biglino G, Giardini A, Hsia T-Y, Figliola R, Taylor AM, Schievano S, et al. Modeling single ventricle physiology: review of engineering tools to study first stage palliation of hypoplastic left heart syndrome. Front Pediatr. 2013;1:31.

39. Hussein N, Lim A, Honjo O, Haller C, Coles JG, Van Arsdell G, et al. Development and validation of a procedure-specific assessment tool for hands-on surgical training in congenital heart surgery. J Thorac Cardiovasc Surg. 2020;160:229-240.e1.

40. Lau IWW, Liu D, Xu L, Fan Z, Sun Z. Clinical value of patient-specific three-dimensional printing of congenital heart disease: Quantitative and qualitative assessments. PLoS One. 2018;13:e0194333.

41. Vigil C, Lasso A, Ghosh RM, Pinter C, Cianciulli A, Nam HH, et al. Modeling Tool for Rapid Virtual Planning of the Intracardiac Baffle in Double-Outlet Right Ventricle. Ann Thorac Surg. 2021;111:2078–83.

42. Illmann CF, Hosking M, Harris KC. Utility and Access to 3-Dimensional Printing in the Context of Congenital Heart Disease: An International Physician Survey Study. CJC Open. 2020;2:207–13.

43. Garekar S, Bharati A, Kothari F, Patil S, Dhake S, Mali S, et al. Virtual three-dimensional model for preoperative planning in a complex case of a double outlet right ventricle. Ann Pediatr Cardiol. 2019;12:295–7.

44. Brun H, Bugge R a. B, Suther LKR, Birkeland S, Kumar R, Pelanis E, et al. Mixed reality holograms for heart surgery planning: first user experience in congenital heart disease. Eur Heart J Cardiovasc Imaging. 2019;20:883–8.

45. Farooqi KM, Uppu SC, Nguyen K, Srivastava S, Ko HH, Choueiter N, et al. Application of Virtual Three-Dimensional Models for Simultaneous Visualization of Intracardiac Anatomic Relationships in Double Outlet Right Ventricle. Pediatr Cardiol. 2016;37:90–8.

46. Yoo SJ, Hussein N, Peel B, Coles J, van Arsdell GS, Honjo O, et al. 3D Modeling and Printing in Congenital Heart Surgery: Entering the Stage of Maturation. Front Pediatr. 2021;9:621672.

47. Yim D, Dragulescu A, Ide H, Seed M, Grosse-Wortmann L, van Arsdell G, et al. Essential Modifiers of Double Outlet Right Ventricle: Revisit With Endocardial Surface Images and 3-Dimensional Print Models. Circ Cardiovasc Imaging. 2018;11:e006891.

48. Yoo S-J, van Arsdell GS. 3D Printing in Surgical Management of Double Outlet Right Ventricle. Front Pediatr. 2017;5:289.

49. Giannopoulos AA, Chepelev L, Sheikh A, Wang A, Dang W, Akyuz E, et al. 3D printed ventricular septal defect patch: a primer for the 2015 Radiological Society of North America (RSNA) hands-on course in 3D printing. 3D Print Med. 2015;1:3.
