# Supplementary material for: Clinical situations for which 3D printing is considered an appropriate representation or extension of data contained in a medical imaging examination: pediatric congenital heart disease conditions
Source: 3D Print Med. 2024 Jan 29;10:3. doi: 10.1186/s41205-023-00199-3 (PMC10823658; doi:10.1186/s41205-023-00199-3)
Supplement: Supplementary file 4 — Supplementary Material 4: Change log detailing additions and amendments from the 2018 appropriateness ratings and this revised manuscript [file 41205_2023_199_MOESM4_ESM.docx]

# Appendix IV: Change Log

## Background

Following increased use of 3D printing in treating congenital heart disease and growing evidence, the 3D Printing Congenital Heart Disease Conditions Voting Group have updated and adjusted appropriateness scores. Significant changes include recategorization of lesions for clarity and changes in scores based on evidence and expert consensus.

Major changes from the previous document are summarized below.

## Lesions

### Atrial Septal Defect (ASD)

ASD was changed from size-based segmentation to be segmented by complexity intended to cover complex variations (e.g., unroofed coronary sinus).

*ASD: Remain Red*

*ASD (additional complexity): Red amended to Green*

### Ventricular Septal Defect (VSD)

VSD was changed from size-based segmentation to be segmented by complexity intended to cover complex variations.

*Lesions “VSD Small” and “VSD Large Noncomplex” combined to “VSD” – No rating change*

*VSD (additional complexity): Yellow to Green*

### Interrupted Aortic Arch

Interrupted Aortic Arch is added to the lesion list and has been refined to reflect two clinical scenarios.

Interrupted Aortic Arch: Red

Interrupted Aortic Arch with LVOT Obstruction: Green

### Partial Anomalous Pulmonary Venous Return (PAPVR)

The rating was amended from Green to Yellow.

### Total Anomalous Pulmonary Venous Return (TAPVR)

The rating was amended from Green to Yellow

### Hypoplastic Left Heart Syndrome (HLHS):

HLHS was segmented to reflect different clinical scenarios.

HLHS (covering initial palliation and s/p stage 1 palliation): Yellow amended to Red

HLHS s/p superior cavopulmonary anastomosis: Remained Yellow

### Shone’s Syndrome

Shone’s Syndrome was removed as a separate clinical scenario.

### Double Inlet Left Ventricle (DILV)

DILV is now reflected under “Single Ventricle (excluding HLHS).”

### Double Outlet Right Ventricle (DIRV)

DILV is now reflected under “Single Ventricle (excluding HLHS).”

### Mitral atresia

Mitral atresia was removed as a separate clinical scenario and is now reflected under HLHS.

### Single Ventricle

Single Ventricle (general) was segmented to reflect different clinical scenarios.

Single Ventricle (excluding HLHS): Yellow amended to Red

Single Ventricle (excluding HLHS) s/p superior cavopulmonary anastomosis: Remained Yellow

### Tricuspid atresia

Tricuspid atresia was removed as a separate clinical scenario and is now reflected under Single Ventricle (excluding HLHS).

### Levo-Transposition of the Great Arteries (L-TGA):

L-TGA is now used for the nomenclature instead of Congenitally Corrected-TGA. The rating was amended from Green to Yellow.

### Dextro-Transposition of the Great Arteries (D-TGA):

D-TGA was segmented to reflect different clinical scenarios.

D-TGA: Green amended to Red

D-TGA with pulmonary stenosis: Remain Green

### Atrioventricular and/or Ventriculoarterial Discordance and (excluding Single Ventricle, TGA, and DORV)

A new classification was created to encompass Atrioventricular and/or Ventriculoarterial Discordance not otherwise accounted for included Double Outlet Left Ventricle, Criss-Cross Heart, etc.

The rating was set at Green
